# Supplementary material for: Super-Toughened Poly(lactic Acid) with Poly(ε-caprolactone) and Ethylene-Methyl Acrylate-Glycidyl Methacrylate by Reactive Melt Blending
Source: Polymers (Basel). 2019 May 1;11(5):771. doi: 10.3390/polym11050771 (PMC6571553; doi:10.3390/polym11050771)
Supplement: Supplementary file 1 [file polymers-11-00771-s001.pdf]

Supplementary Information

Super Toughened Poly(lactic Acid) with Poly(ε-caprolactone) and Ethylene-Methyl Acrylate-Glycidyl Methacrylate by Reactive Melt Blending

Ao-Lin Hou and Jin-Ping Qu *

National Engineering Research Center of Novel Equipment for Polymer Processing, Key Laboratory of Polymer Processing Engineering, Ministry of Education, Guangdong Provincial Key Laboratory of Technique and Equipment for Macromolecular Advanced Manufacturing, School of Mechanical and Automotive Engineering, South China University of Technology, Guangzhou 510640, China; houaolin2016@163.com

* Correspondence: jpqu@scut.edu.cn; Tel.: +86-(020)-8711114

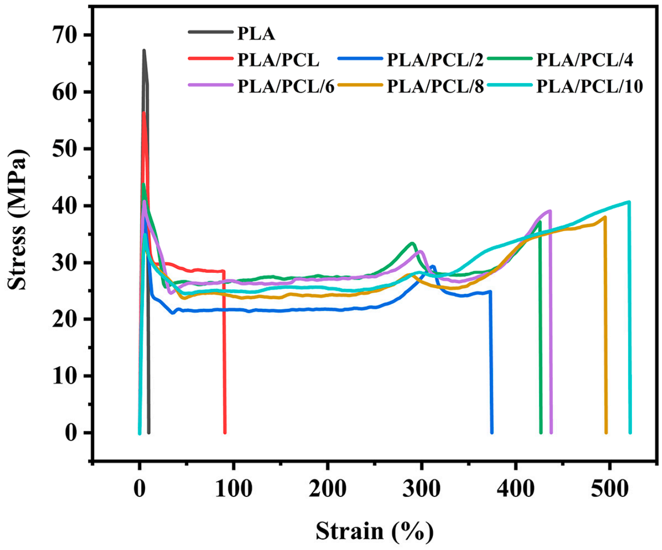

Figure S1. XRD patterns of PLA/PCL with different addition of EMA-GMA.

Remarkably, a strange phenomenon appeared in the stress-strain curves of PLA/PCL/EMA-GMA blends. As we all known, four parts which are elastic deformation, yielding, developing large deformation, and strain-stiffing comprise the typical stress-strain curves of polymers. However, as shown in the Figure S1, second elastic and yielding deformation appeared in the developing large deformation stage of the ternary blends. The size of the second yielding was influenced by the addition amount of EMA-GMA. Regularly, the positions of the second yielding were almost the same, and the level became smaller and smaller with the increase of EMA-GMA contents until it almost disappeared at the addition of 8 or 10 phr EMA-GMA. This phenomenon hasn’t been found in numerous studies of PLA/EMA-GMA blends [1-2], but in this work, it appeared in all samples of ternary blends and one sample of PLA/PCL blend whose elongation at break exceeded 110% (refer to Figure S2).

The tensile stress would transfer to the dispersed PCL phase of blends under a strong interfacial adhesion. When tensile strain exceeded a certain level, the tensile deformation of dispersed PCL phase would emerge. The elongation of the amorphous part of the PCL phase

was limited by the crystalline zone, which resulted in the increase of tensile stress, while the orientation of the chain-folded lamellae remained unchanged. With the tension increased, the lamellae were fragmentized into checkerboard-like arrangement causing the yielding deformation [3]. According to the analysis, PCL phase had poor interfacial adhesion with PLA matrix, therefore the tensile deformation of PCL dispersed phase appeared only in a few samples of PLA/PCL blend whose elongation at break exceeded a certain level. After EMA-GMA was added, the introduction of EMA-GMA reduced the degree of crystallinity of PCL phase presenting as the decrease of Young's modulus and yield strength in the second elastic and yielding deformation, and then strengthened the continuity of the stress transfer between the PLA matrix and dispersed PCL phase due to the enhanced interfacial adhesion by reaction between the PCL and EMA-GMA. Therefore, the tensile deformation caused by the elongation of PCL phase was gradually decreased with the increase of the EMA-GMA contents.

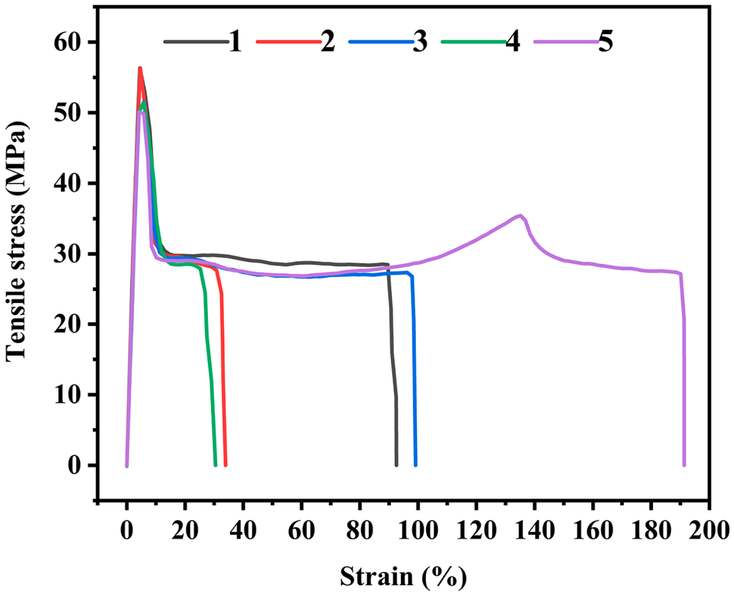

Figure S2. Stress-strain curves of all the 5 samples of PLA/PCL formulation

References

1. Li, X.; Kang, H.; Shen, J.; Zhang, L.; Nishi, T.; Ito, K.; Zhao, C.; Coates, P., Highly toughened polylactide with novel sliding graft copolymer by in situ reactive compatibilization, crosslinking and chain extension. *Polymer* **2014,** *55* (16), 4313-4323, DOI: 10.1016/j.polymer.2014.06.045.
2. Zhang, K.; Nagarajan, V.; Misra, M.; Mohanty, A. K., Supertoughened renewable PLA reactive multiphase blends system: phase morphology and performance. *ACS Appl Mater Interfaces* **2014,** *6* (15), 12436-48, DOI: 10.1021/am502337u.
3. Kamal, T.; Shin, T. J.; Park, S. Y., Uniaxial Tensile Deformation of Poly(ε-caprolactone) Studied with SAXS and WAXS Techniques Using Synchrotron Radiation. *Macromolecules* **2012,** *45* (21), 8752-8759, DOI: 10.1021/ma301714f.
